# Myeloarchitectonic plasticity in elite golf players' brains

**DOI:** 10.1002/hbm.25860

**Published:** 2022-04-14

**Authors:** Xueyun Shao, Daiyi Luo, Yulong Zhou, Zhuoni Xiao, Jinjian Wu, Li Hai Tan, Shijun Qiu, Di Yuan

**Affiliations:** ^1^ School of Sports, Shenzhen University Shenzhen China; ^2^ Shenzhen Institute of Neuroscience Shenzhen China; ^3^ Department of Psychology University of Edinburgh Edinburgh UK; ^4^ Department of Radiology First Affiliated Hospital of Guangzhou University of Chinese Medicine Guangzhou China; ^5^ Guangdong‐Hongkong‐Macau Institute of CNS Regeneration Jinan University Shenzhen China; ^6^ Neuroscience and Neurorehabilitation Institute, University of Health and Rehabilitation Sciences Qingdao Shandong China; ^7^ Department of Psychology The Chinese University of Hong Kong Hong Kong SAR China

**Keywords:** qMRI, microstructure, golfing proficiency, temporal pole

## Abstract

Human neuroimaging studies have demonstrated that exercise influences the cortical structural plasticity as indexed by gray or white matter volume. It remains elusive, however, whether exercise affects cortical changes at the finer‐grained myelination structure level. To answer this question, we scanned 28 elite golf players in comparison with control participants, using a novel neuroimaging technique—quantitative magnetic resonance imaging (qMRI). The data showed myeloarchitectonic plasticity in the left temporal pole of the golf players: the microstructure of this brain region of the golf players was better proliferated than that of control participants. In addition, this myeloarchitectonic plasticity was positively related to golfing proficiency. Our study has manifested that myeloarchitectonic plasticity could be induced by exercise, and thus, shed light on the potential benefits of exercise on brain health and cognitive enhancement.

## Graphical abstract

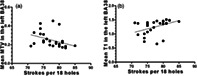

## 1 INTRODUCTION

The adult human brain shows structural plasticity in response to environmental demands. Previous neuroimaging studies demonstrated that the macroscopic structure of healthy adult human brains exhibits stimulus‐dependent alteration (B. Draganski et al., 2002; Mechelli et al., 2004; Stein et al., 2012). For example, experienced London taxi drivers, compared with control subjects who did not drive taxis, had a significantly greater gray matter volume in the posterior hippocampus which stores spatial representations of the environment (Maguire et al., 2000). And the volume of the posterior hippocampus was positively correlated with the amount of time spent as a taxi driver. Short‐term training is also known to quickly induce changes in the anatomical structure of healthy human brain indexed by gray matter. In a voxel‐based morphometry (VBM) study, adults who were inexperienced in juggling showed a significant transient bilateral expansion in gray matter in the mid‐temporal area and in the left posterior intraparietal sulcus after a 3‐month training on a classic three‐ball cascade juggling routine (Bogdan Draganski et al., 2004). In another VBM study, 2‐h training on learning newly defined and named subcategories of the universal categories “green” and “blue” increased participants' volume of gray matter in V2/3 of the left visual cortex, a region known to mediate color vision (Kwok et al., 2011).

Exercise, as a valid cognitive‐enhancement strategy, has been demonstrated to influence structural plasticity (Haeger et al., 2019). Studies utilizing rodent models showed that exercise activated mechanistic target of rapamycin (mTOR) which improved neuron activities, axonal myelinations, and synaptic transmissions in mice after 21‐day chronic treadmill running exercise (Chen et al., 2019). Leiter et al. (2022) separated mice into the control group and exercise group which performed wheel running for 4 days and found that selenium metabolism was required for the exercise‐induced enhancement in adult hippocampal neurogenesis. Neuroimaging research into the human brain revealed that a 2‐week cardiovascular exercise (CE) could improve the learning rate in a subsequent motor task, in which the learning improvement was conveyed by CE‐induced changes in white matter in frontotemporal fiber tracts of the human brain (Lehmann et al., 2020). An 8‐week training of Tai Chi Chuan or general aerobic exercise, compared with no exercise intervention, increased participants' gray matter volume in the left precuneus implying the benefits of exercise in memory retrieval, meditation, and attention to depression (Cui et al., 2019). In addition, studies that compared athletes with nonathletes also suggested the plastic change of macro‐level structural brain induced by motor skill acquisition. For example, diving players showed significantly increased gray matter density in the thalamus and left precentral gyrus compared with control subjects (Wei et al., 2009), and basketball players showed morphological enlargement in the striatum compared with controls (Park et al., 2011). A previous study found that even undergoing 40 h of golf practice as a novice would increase macro‐level gray matter volume in the left primary motor cortex and the left ventral premotor cortex (Bezzola et al., 2011).

Does exercise affect cortical changes at finer‐grained level, for example, the myelination structure of the living human brain? To answer this question, we examined 28 elite golf players in comparison with control participants with a novel neuroimaging technique—quantitative magnetic resonance imaging (qMRI). Golf is a challenging sporting pursuit that requires a range of physically demanding movement patterns which encompass motor, visual, mental, tactical, and technical attributes (Lennon, 1998; Smith, 2007). Sensorimotor rhythm activity is known to be associated with sports performance such as golf putting (Cheng et al., 2015). Stroke patients who received a 10‐week golf training showed a significant improvement in the visual mental rotation task compared to the control group (Schachten & Jansen, 2015). Shimada et al. (2018) found improvements in immediate and delayed logical memory after a 24‐week golf training with healthy older adults. Learning to play golf over 22 weeks improved cognitive functions and the regulation of dysregulation of the kynurenine pathway that affects cognition in elderly people (Stroehlein et al., 2021).

The neuroimaging measures used in previous studies are qualitative because they are derived from uncalibrated T1‐weighted images, which are sensitive to multiple features of tissue organization and microstructure (Mezer et al., 2013). To quantitatively evaluate microstructural properties in vivo, in the present study we employed the qMRI to compute the brain macromolecular tissue volume (MTV) and quantitative T1, which linearly contributes to iron and myelin concentrations (Stüber et al., 2014). As cell membranes and proteins account for the majority of brain macromolecules, MTV provides a valid approximation of myelin volume (Berman et al., 2018; Luo et al., 2019; Yuan et al., 2021). Developmental decrease of T1 is thought to result from microstructural proliferation such as dendrite development, myelination, and oligodendrocytes (Gomez et al., 2017; Luo et al., 2019; Yuan et al., 2021). We used MTV and quantitative T1 to determine the microstructural variation in the brain tissue of both golf players and control participants. Hypothetically, golf players may show more proliferated microstructure than the controls, due to years of demanding training, and the myeloarchitectonic proliferation may be associated with golfing proficiency.

## 2 MATERIALS AND METHODS

### 2.1 Participants

A total of 55 adults were recruited in the current experiment including 28 golf players (mean age: 24.61 years with *SD* at 5.20 years, 20 males and 8 females) and 27 nonplayers (mean age: 24.56 years with *SD* at 4.68 years, 19 males, and 8 females). The two groups of participants showed comparable cognitive abilities measured by the reading task (Yuan et al., 2021), the majority function task (MFT, Wu et al., 2016), and the visual‐motor integration task (VMI, Spencer & Kruse, 2013) (*p* > .05 in two‐sample *t* tests, Table 1). The previous average golf score for each golf player was recorded on a par‐72 course. The average golf score in the golf group was 77.68 strokes/18 holes (ranging from 71 to 85) and this group of participants was termed the elite golf players. All the participants were right‐handed as measured by the Handedness Inventory (Snyder & Harris, 1993). They were physically and neurologically healthy with normal or correct‐to‐normal vision. They gave informed consent before the experiment and were each paid after the experiment. The study was approved by the ethics committee at the Shenzhen Institute of Neuroscience and in accordance with the Declaration of Helsinki.

**TABLE 1:** Behavioral performance of participants

	Mean		Standard deviation		Minimum		Maximum		P value
	Golf player	Nonplayer	Golf player	Nonplayer	Golf player	Nonplayer	Golf player	Nonplayer	
Score of VMI task	91	94.07	9.96	14.74	66	50	107	107	.367
Accuracy of reading task	83.10%	86.53%	9.20%	5.32%	57.46%	72.68%	94.87%	95.93%	.098
Accuracy of MFT	88.24%	86.27%	5.70%	5.52%	68.75%	70.83%	97.92%	95.83%	.197

Abbreviations: MFT, majority function task; VMI, visual‐motor integration.

### 2.2 Measures

#### 2.2.1 The visual‐motion localizer task

Since golf play is highly related to visual motor skills, to identify the visual‐ and motor‐related brain regions, we adopted a passive video watching task for localizing purposes in this experiment. All participants were asked to passively watch four 16‐s videos, each showing a golf player's swing performance. After each video, a 16‐s rest section was presented during which participants were asked to remain relaxed and fixate at the crosshair in the center of the screen. The stimuli were presented via an LCD projector system during functional magnetic resonance imaging (fMRI).

#### 2.2.2 fMRI data acquisition

All images were acquired on a Siemens (Munich, Germany) 3T Prisma scanner using a standard 64‐channel head coil. Functional volumes were acquired using a multiple slice T2*‐weighted echo planar imaging (EPI) sequence with the following parameters: repetition time = 2,000 ms; echo time = 30 ms; flip angle = 90°; matrix dimensions = 64 × 64; field of view = 100 mm; slice thickness = 3.5 mm; number of slices = 33. High‐resolution three dimensional T1‐weighted images were acquired using the magnetization‐prepared rapid gradient‐echo (MPRAGE) sequence with the following parameters: repetition time (TR) = 2,530 ms; echo time (TE) = 2.98 ms; inversion time = 1,100 ms; flip angle = 7°; field of view (FOV) = 256 mm × 224 mm; matrix size = 224 × 256, interpolated to 448 × 512, 192 sagittal slices; slice thickness = 1.0 mm; voxel size = 0.5 × 0.5 × 1 mm^3^.

#### 2.2.3 Quantitative MRI data acquisition

Quantitative MRI measurements were guided by the protocols in Mezer et al. (2016); Mezer et al. (2013) and Oishi et al. (2018). The whole‐brain MRI data was measured by 3T Siemens Magnetom Prisma scanner (Siemens Healthcare, Erlangen, Germany) with a 64‐channel head coil. The quantitative MTV and T1 values were measured from spoiled gradient echo (SPGE) images with flip angles of 4°, 10°, 20°, and 30° (TR = 12 ms, TE = 2.41 ms, FOV = 225 mm, matrix size = 224 × 244) at an in‐plane resolution of 1 × 1 mm^2^ with a slice thickness of 1 mm. Four spin‐echo inversion recovery (SEIR) images were also scanned, with an EPI read‐out, a slab inversion pulse, and spectral fat suppression, to remove field inhomogeneities. The images were collected with inversion times of 50, 200, 400, 1200, and 2400 ms (TE = 49 ms, TR = 3000 ms, FOV = 272 mm, matrix size = 122 × 122) at an in‐plane resolution of 2.2 × 2.2 mm^2^ with a slice thickness of 4 mm.

### 2.3 Data analyses

#### 2.3.1 MRI data analyses

Preprocessing and statistical analyses of imaging data were performed with SPM12 (Wellcome Department of Imaging Neurosciences, University College London, UK, http://www.fil.ion.ucl.ac.uk/spm), a toolbox running on Matlab R2018b.

In the preprocessing, all functional images obtained during the localizer task were first corrected for slice timing with the middle slice in the acquisition order as a reference and realigned to the mean of functional scans to remove movement artifact. The T1 image was coregistered to a mean functional image, then segmented. Functional images were then normalized to the Montreal Neurological Institute (MNI) space with the deformation fields produced after segmentation. Voxel size was resampled to 2 × 2 × 2 mm^3^. Isotropic Gaussian kernel with 6 mm full‐width at half maximum was used for spatial smoothing.

Individual activation maps were then generated by using the general linear model (GLM). Stimulus onsets and duration of conditions were convolved with the canonical hemodynamic response function. Realignment parameters were included in the model to regress out movement‐related variance. Each time series was high‐pass filtered with a cutoff period set at 128 s to remove low‐frequency drifts. GLM was used to produce contrast maps comparing the video watching blocks and the rest blocks at both individual and group levels. One‐sample *t* tests were performed for the whole brain activation of the contrast of interest [*p* < .05, false discovery rate (FDR) corrected, extent threshold = 10 voxels] for all the participants (i.e., golf players and nonplayers).

#### 2.3.2 QMRI data analysis

Both SPGE images and SEIR images were processed by using the mrQ software package (https://github.com/mezera/mrQ) to generate maps of MTV and quantitative T1 for each participant. SPGE images and low‐resolution unbiased T1 maps derived from SEIR images were combined (Barral et al., 2010) to estimate the unbiased T1 maps and proton density maps (Fram et al., 1987). MTV maps were calculated from proton density maps. MTV quantified the nonwater volume in each voxel and the complementary volume was water (cerebrospinal fluid was approximated to water). T1‐weighted images were transformed into the cortical surface using Freesurfer 6.0 recon‐all procedure (Reuter et al., 2012). They were spatially matched with MTV maps and had excellent gray/white matter contrast.

Following the previous research (Gomez et al., 2017; Luo et al., 2019), the regions of interest (ROIs) in our study were selected based on the anatomical location of the functional peak maxima of the localizer task. Destrieux atlas in FreeSurfer was used to automatically label the cortex (Destrieux et al., 2010). The boundaries of the labels are customized to each participant based on curvature statistics stored in the atlas. For each participant, the “mri_convert” command was used to convert the individual labeled template into the native MTV space. Then, mean MTV and T1 values across voxels within ROI for each participant were computed.

#### 2.3.3 Statistical analysis

Two‐sample *t* tests were performed to examine the difference of microstructure values (MTV and T1) between golf player and nonplayer groups. Because we assumed that the microstructural proliferation of golf players would be greater than that of nonplayers, the *t* tests were used with one tailed (Evans et al., 2020; Li et al., 2020). False discovery rate corrections were applied across ROIs. Then, partial correlations were conducted to examine the relationship between microstructural value and golfing proficiency (i.e., golf score) with age as the covariant.

## 3 RESULTS

### 3.1 Behavioral performance

**FIGURE 1:**
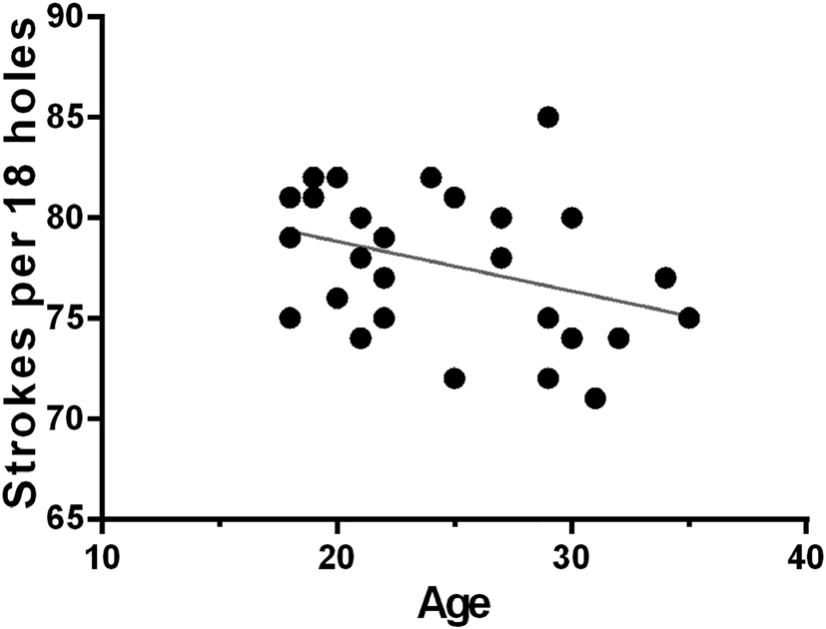
Behavioral performance of participants. Pearson correlation between the golf score (in terms of strokes per 18 holes) and age in golf player group

In the golf player group, the golf score was marginally negatively related to age suggesting that the golf performance is higher as the golf players get older (*r* = −.357, *p* = .0619, Figure 1).

### 3.2 Regions activated in the localizer task

**FIGURE 2:**
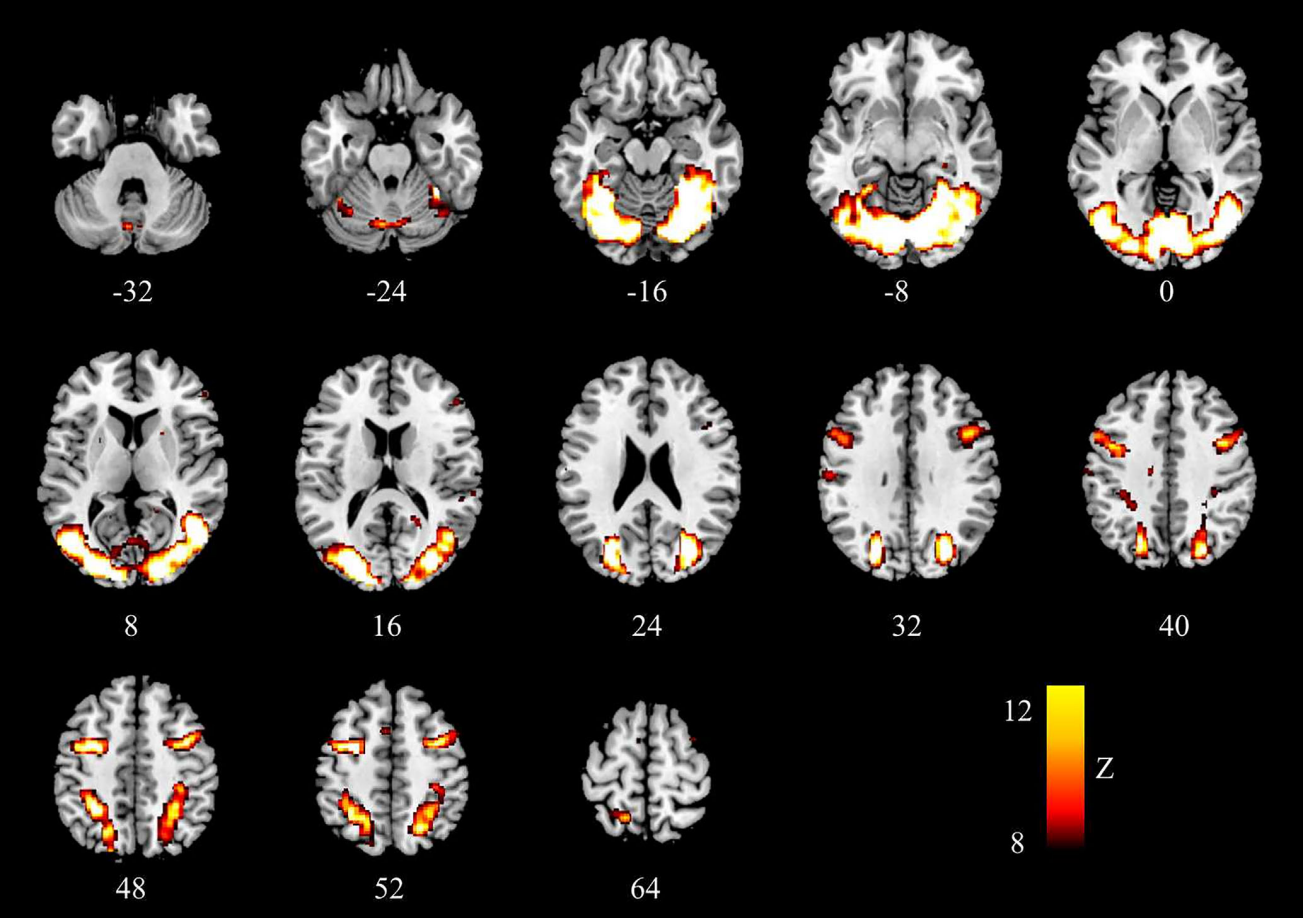
Brain regions that were significantly activated during the video watching localizer blocks compared to the rest blocks (whole brain FDR correction at *p* < .05). FDR, false discovery rate

During the localizer task (relative to the fixation baseline), six regions were strongly activated in the whole‐brain based analysis averaged across the golf player group and the nonplayer group (survived at *p* < .05, FDR corrected, extent threshold = 10 voxels): left cerebellum (MNI: −4, −78, −6), left cuneus (BA17, MNI: −26, −80, 16), right cerebellum (MNI: 20, −66, −12), left superior temporal gyrus (BA38, MNI: −40, 16, −28; −52, 12, −8), left inferior frontal gyrus (BA47, MNI: −42, 20, −18), left caudate (MNI: −20, −30, 22) (Table 2 and Figure 2). Since cerebellum and caudate are not defined in the Destrieux atlas, they were excluded from further analysis (Destrieux et al., 2010). Two cortical regions which are responsible for visual processing were selected as ROIs: left BA17 and left BA38 (Dupont et al., 1994; Ungerleider et al., 1982; Vanni et al., 2001; Zhuo et al., 2003). They were identified as left cuneus and left temporal pole in the Destrieux atlas.

**TABLE 2:** Local maxima of activation during the localizer task compared to resting baseline (whole brain FDR correction at *p* < .05)

Regions activated	BA	Coordinates (MNI)			*Z‐value*	Voxels
		X	Y	Z		
Left cuneus	17	−26	−80	16	Inf	66541
Left cerebellum		−4	−78	−6	Inf	
Right cerebellum		20	−66	−12	Inf	
Left superior temporal gyrus	38	−40	16	−28	4.93	264
Left superior temporal gyrus	38	−52	12	−8	3.78	
Left inferior frontal gyrus	47	−42	20	−18	4.78	
Left caudate		−20	−30	22	3.24	37

Abbreviation: BA, Brodmann Area; FDR, false discovery rate; MNI, Montreal Neurological Institute.

### 3.3 Mean microstructural value in each ROI

**FIGURE 3:**
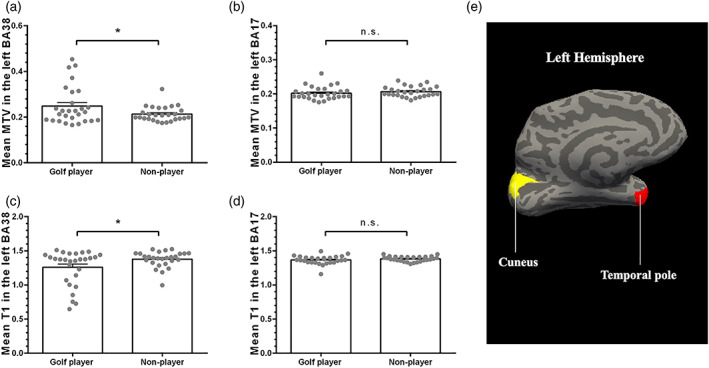
The difference of microstructure values between golf player group and nonplayer group. There were significant differences in mean MTV (a) and mean T1 (c) in the left BA38 between the golf player group and the nonplayer group. However, there was no significant difference in mean MTV (b) and mean T1 (d) in the left BA17 between the golf player group and the nonplayer group. (e) The location of selected ROIs on an inflated cortical surface (left, medial side). The yellow area represents the cuneus, and the red area represents the temporal pole. MTV, macromolecular tissue volume; ROI, regions of interest

The mean MTV value in the left BA38 is significantly larger in the golf player group than the nonplayer group (*t*_53_ = 2.046, *p*_corrected_ = 0.023; for the golf player group: *M* = 0.248, *SD* = 0.083; for the nonplayer group: *M* = 0.213, *SD* = 0.033, Figure 3a), indicating that golf playing is associated with better development of the brain at the micro myelination level. No significant difference of the mean MTV value in the left BA17 was found between golf player group and nonplayer group (*t*_53_ = −0.008, *p*_corrected_ = 0.497; for the golf player group: *M* = 0.206, *SD* = 0.016; for the nonplayer group: *M* = 0.206, *SD* = 0.015, Figure 3).

The mean T1 value in the left BA38 was significantly smaller in the golf player group than in the nonplayer group (*t*_53_ = −2.218, *p*_corrected_ = 0.031; for the golf player group: *M* = 1.258, *SD* = 0.257; for the nonplayer group: *M* = 1.378, *SD* = 0.117, Figure 3c), again suggesting that golf play is associated with better development of the brain's myeloarchitectonic structure. No significant difference of the mean T1 value in the left BA17 was seen across the two groups (*t*_53_ = −1.145, *p*_*corrected*_ = 0.258; for the golf player group: *M* = 1.366, *SD* = 0.117; for the nonplayer group: *M* = 1.382, *SD* = 0.038, Figure 3d).

### 3.4 The microstructure–behavior relationship

**FIGURE 4:**
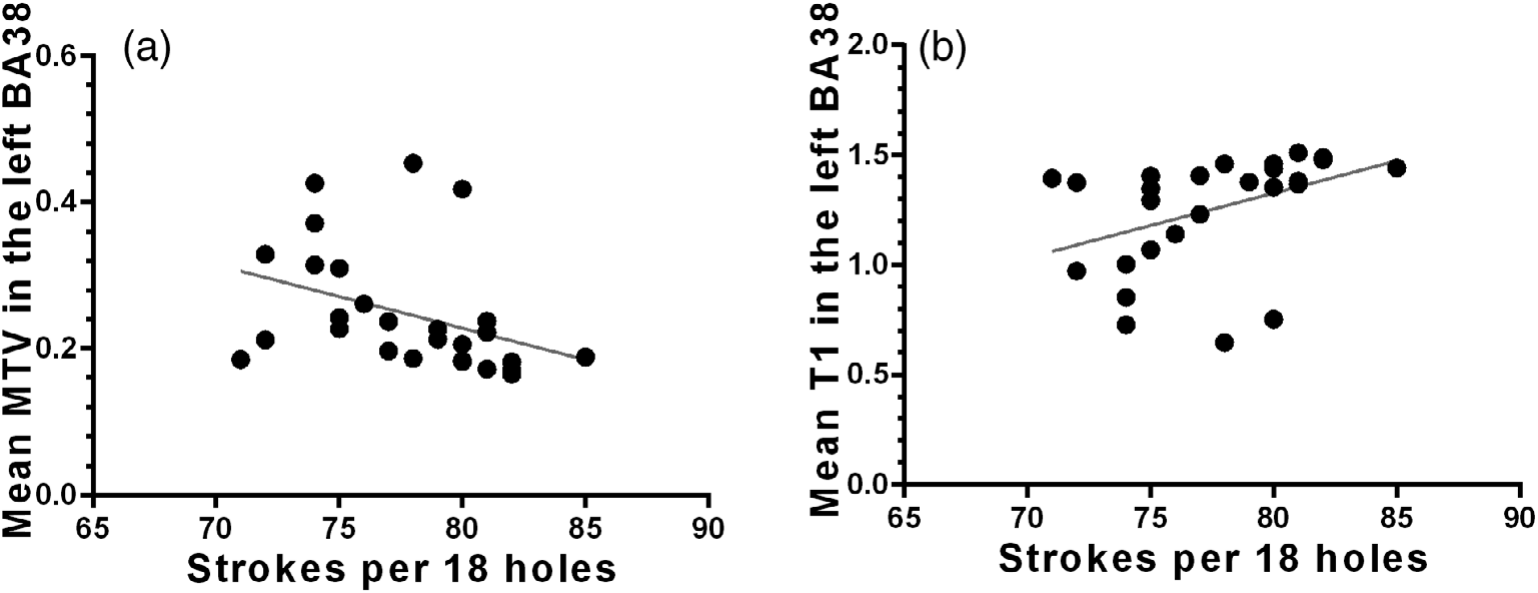
The relationship between golf score and the microstructural value (mean MTV in panel (a), and mean T1 in panel (b) in the left BA38 under the control of age effect. MTV, macromolecular tissue volume

Partial correlations were conducted to examine the relationship between the microstructure of the left BA38 and the golf score under the control of age effect. In the golf player group, the mean MTV was negatively related to golf score (*r* = −.382, *p* = .049, Figure 4a); the mean T1 was positively related to golf score (*r* = .404, *p* = .037, Figure 4b). These results suggested that the microstructure proliferation was positively related to golfing proficiency.

## 4 DISCUSSION

In the current study, we used the qMRI technique to examine the microstructural proliferation of two visual processing brain regions (i.e., left cuneus and left temporal pole which were defined with reference to activate brain sites in our localizer task) of golf players and nonplayers. We found that the microstructure in the left temporal pole of golf players was more proliferated than that of nonplayers. Moreover, such microstructural plasticity shown in golf players was positively related to golfing proficiency.

Our results are in good consistence with previous findings of brain structural change induced by exercise (Cui et al., 2019; Haeger et al., 2019; Lehmann et al., 2020; Park et al., 2011; Wei et al., 2009). Crucially, we found a positive relationship between the microstructural proliferation and golfing proficiency of golf players. According to the record, about 10,000 practice hours are necessary to become a professional golfer (Canadian, 2007). Previous findings had demonstrated that the macrostructural plasticity of golfers was associated with their training intensity (Bezzola et al., 2011; Jäncke et al., 2009). Our results extend the knowledge of the cortical plasticity to the myeloarchitectonic structure and by showing the relationship between the microstructural proliferation and golfing proficiency of golf players from the microstructural perspective. Note that such a correlation found in our study does not ensure a causal relationship. Further studies should investigate how the structural plasticity may be developed by exercise.

Playing golf comprises adaptations in visuospatial functions which correspond well to the regions showing microstructural plasticity in our study. The ventromedial part of temporal pole was predominantly functional connected to higher‐level visual regions (Pascual et al., 2015). Specifically, the exclusive connectivity between the temporal pole and the vestibular nuclei suggested the role of the temporal pole in eye‐movement control. The temporal pole was also connected to the anterior hippocampus, known to mediate vestibular processing in humans (Hüfner et al., 2011). These findings imply that the temporal pole might modulate the vestibular system to change the level of vestibular control over eye movements, according to prior knowledge about requirements for interaction with specific objects identified visually (Pascual et al., 2015). Moreover, the left temporal pole is an important region for high‐level sensory representations with semantic information (Glosser et al., 2003; Olson et al., 2007; Snowden et al., 2004). The proliferated microstructure of elite golfers in the temporal pole also implies the benefit of exercise on cognitive enhancement. As previous studies demonstrated, learning and memory abilities can be improved by a few weeks of training (Cui et al., 2019; Lehmann et al., 2020). How the length and frequency of golf exercise influence microstructural plasticity should be examined in further research.

Although a number of studies found that plastic changes of structural brain can be induced by exercise, the brain structures enhanced across the studies focusing on different exercises seem to be overlapping and distinct (Bezzola et al., 2011; Cui et al., 2019; Park et al., 2011; Wei et al., 2009). The ROIs in our study might be golf‐specific because the localizer task narrowed down our focus to brain regions that are highly related to golf skills. However, the effect of golf on the human brain at the microstructural level seen in our study should be generalizable to other exercises involving similar skills (e.g., visual motion skills) because of the plasticity of human brains. Further studies are needed to elaborate the causal relationship between exercise and microstructural brain plasticity.

## 5 CONCLUSION

The current study applied the qMRI technique to compare the myeloarchitectonic proliferation in the brain of elite golf players and nonplayers. Golf players showed higher myeloarchitectonic proliferation in the left temporal pole than nonplayers. Moreover, this myeloarchitectonic proliferation is positively related to golfing proficiency. The findings highlighted the potential benefits of exercise on brain health and cognitive enhancement.

## CONFLICT OF INTEREST

The authors declare no competing financial interests.

## Data Availability

The data that support the findings of this study are available from the corresponding authors, Di Yuan and Shijun Qiu, upon reasonable request.
